# The Child as a Surrogate for Diagnosis of Lupus in the Mother

**DOI:** 10.1155/2017/8247591

**Published:** 2017-03-13

**Authors:** Olufemi O. Adelowo, Kenneth A. Ohagwu, Ejiehi E. Aigbokhan, Richard O. Akintayo

**Affiliations:** ^1^Rheumatology Unit, Department of Internal Medicine, Lagos State University Teaching Hospital, Ikeja, Nigeria; ^2^Rheumatology Unit, Internal Medicine Department, Federal Medical Centre, Umuahia, Nigeria; ^3^Rheumatology Unit, Department of Internal Medicine, University of Benin Teaching Hospital, Benin City, Nigeria; ^4^Rheumatology Unit, Department of Internal Medicine, University of Ilorin Teaching Hospital, Ilorin, Nigeria

## Abstract

*Introduction.* Neonatal lupus erythematosus (NLE) is an acquired disease of the newborn caused by transplacental transfer of maternal anti-Ro/SSA, anti-La/SSB, and infrequently anti-U1 RNP antibodies.* Methodology.* This is a case report of a male infant delivered via Caesarean section at 36-week gestation following detection of fetal bradycardia during routine antenatal clinic visit.* Results.* The mother was seropositive for antinuclear antibody (ANA) and anti-Ro/SSA and had elevated erythrocyte sedimentation rate. The baby was positive for ANA, extractable nuclear antigen (ENA), and anti-Ro/SSA. Pediatric echocardiography was abnormal and electrocardiography confirmed complete heart block.

## 1. Introduction

Neonatal lupus erythematosus (NLE) is an acquired disease of the newborn caused by transplacental transfer of maternal anti-Ro/SSA, anti-La/SSB, and anti-U1 RNP antibodies, usually between 12–16 weeks of gestation. It is a rare disease though it had been previously reported in Nigeria [1]. It occurs in 1 of every 20,000 live births in the USA. Mothers of the infants may be asymptomatic but may go on to develop features of connective tissue disease, especially systemic lupus erythematosus and Sjogren's syndrome [2, 3]. NLE can be divided into cardiac and noncardiac NLE. Noncardiac manifestations are usually transient and not life-threatening whereas the cardiac manifestations are associated with increased morbidity and mortality. Noncardiac NLE is associated with prenatal exposure to high titers of anti-La/SSB antibodies while cardiac NLE is associated with high titers of anti-Ro/SSA antibodies. Anti-U1 RNP is not associated with cardiac NLE. Genetic polymorphism is associated with the development of complete heart block (CHB) [4].

## 2. Case Presentation

A 32-year-old Nigerian female, who was referred to a private rheumatology clinic in Lagos Nigeria, had presented at 36-week gestation for routine antenatal care when, during examination, fetal heart rate was found to be 42 beats per minute. She subsequently had an Emergency Caesarean section done with delivery of a live male neonate weighing 2.58 kg. The neonate was normal in appearance and had good APGAR score. He had bradycardia (40 beats per minute) and pansystolic murmur. He was reviewed by a pediatric cardiologist. Electrocardiogram and echocardiograph done revealed a complete heart block, patent foramen ovale, and patent ductus arteriosus. He had no other relevant physical findings on examination and no cutaneous lesion.

The mother developed postoperatively a fever with chills and rigor. Investigations had revealed an elevated erythrocyte sedimentation rate of 103 mm/hour. In view of the fetal bradycardia, maternal lupus was suspected and serology requested with positive antinuclear antibody (ANA) with a titre of 1 : 320.

The mother was then referred by the Obstetrician to our facility, 9 days after delivery. She had no history or examination findings suggestive of any connective tissue disease, including SLE. There was no known family history of SLE, including her first child.

Investigations requested further confirmed the mother was positive for ANA (1 : 320) and anti-Ro/SSA (124 iu/ml) while the neonate was positive for extractable nuclear antigen (ENA), anti-Ro/SSA (58 iu/ml) antibody, and ANA (1 : 160). The mother and baby were negative for anti-dsDNA, anti-La/SSB, and U1RNP.

The mother was commenced on hydroxychloroquine while the neonate was tried with dexamethasone administered daily, intramuscularly with no improvement in the heart rate. His cardiac activity was closely monitored. He was also referred to a pediatric cardiologist/cardiothoracic surgeon for pacemaker placement. Subsequent echocardiography done did not reveal any structural abnormality, suggesting closure of the patent foramen ovale and the patent ductus arteriosus. Child is under observation and may need a cardiac pacemaker at four years.

## 3. Discussion

NLE is an acquired disease resulting from the transplacental transfer of maternal anti-Ro/SSA, anti-La/SSB, and anti-U1 RNP antibodies to the fetus around the 12th to 16th week of gestation [5]. The presence of these antibodies is associated with systemic lupus erythematosus and Sjogren's syndrome and this means NLE can occur in either of these conditions affecting the mother as well as other autoimmune diseases. It has also been reported in rheumatoid arthritis [6].

Conduction abnormality is the commonest manifestation of cardiac NLE. The peak onset for diagnosis of congenital heart block in utero is between the 18th and 24th weeks of gestation. Heart block may be first-degree, second-degree, and complete heart block. Other conduction abnormalities include right bundle branch block, sinus bradycardia, and prolonged QT interval. Mechanical and structural abnormalities may also be seen in cardiac NLE. The mechanical abnormalities include cardiomyopathy, congestive heart failure, endomyocardial fibroelastosis, and valvular lesions. Structural abnormalities include atrial septal defects, ventricular septal defects, patent ductus arteriosus, and patent foramen ovale [7]. Our patient had initially presented with a patent ductus arteriosus and patent foramen ovale.

Diagnosis of NLE is made when a newborn of a mother with antibodies to SSA/Ro, SSB/La, or U1 RNP develops complete heart block (and or other cardiac manifestations), typical rash, and hepatic, neurologic, or hematologic manifestations. Diagnosis, like in this case, can also be made in the presence of fetal bradycardia in a mother with positive serology. Prenatal testing is done only for mothers with a high risk like women with SLE, Sjogren's syndrome, and undifferentiated connective tissue disease. It is also recommended in mothers with a history of NLE in previous children. The overall recurrence rate of cardiac NLE is 19% [2].

Prevention of complete heart block can be achieved by treating the mothers with fluorinated corticosteroids. These have the ability to cross the placenta where they prevent fibrosis in the fetal heart. They may improve cardiac conduction in fetuses with first-degree or second-degree heart block. Corticosteroids have no effect on fetuses/neonates with complete heart block. Serial echocardiography should be done from the 16th week of gestation for any fetus with anti-Ro/SSA positive mothers. Cardiac pacemakers are required for patients with complete heart block [3]. Maternal hydroxychloroquine use may reduce the occurrence of NLE in subsequent pregnancies [2].

## 4. Conclusion

This case is presented to draw attention to the possibility of diagnosing SLE in a mother whose baby had presented with complete heart block from SLE.

## Additional Points

*Learning Points.* (1) This report serves to highlight neonatal lupus erythematosus as a cause of neonatal congenital complete heart block in babies born of yet asymptomatic mothers. (2) There is the need to screen babies with congenital heart block and their mothers for antibodies that can cause heart block.
